# Guanidinoacetic Acid as a Dietary Additive for Lambs: A Meta-Analysis on Performance, Antioxidant Status, Nutrient Digestibility, Ruminal Fermentation and Meat Quality

**DOI:** 10.3390/biology15151288

**Published:** 2026-08-04

**Authors:** José Felipe Orzuna-Orzuna, Juan Eduardo Godina-Rodríguez, Germán David Mendoza-Martínez, Gabriela Vázquez-Silva, Pablo Benjamín Razo-Ortiz, Cesar Díaz-Galván, Nallely Sánchez-López, Pedro Abel Hernández-García

**Affiliations:** 1 Unidad Regional Universitaria de Zonas Áridas, Universidad Autónoma Chapingo, Bermejillo CP 35230, Mexico; jforzuna@gmail.com; 2 Instituto Nacional de Investigaciones Forestales, Agrícolas y Pecuarias, Campo Experimental Valle de Apatzingán. Carretera Apatzingán-Cuatro Caminos km 17.5, Parácuaro CP 60781, Michoacán, Mexico; godina.juan@inifap.gob.mx; 3 División de Ciencias Biológicas y de la Salud, Departamento de Producción Agrícola y Animal, Universidad Autónoma Metropolitana Xochimilco, Mexico City CP 04960, Mexico; gmendoza@correo.xoc.uam.mx (G.D.M.-M.); mvzrazo@gmail.com (P.B.R.-O.); cesarwardi14@gmail.com (C.D.-G.); 4 Departamento del Hombre y su Ambiente, Universidad Autónoma Metropolitana—Xochimilco, Mexico City CP 04960, Mexico; gavaz@correo.xoc.uam.mx; 5 SECIHTI Programa de Investigadoras e Investigadores por México-Universidad Autónoma Metropolitana Xochimilco, Mexico City CP 04960, Mexico; nsanchezl@correo.xoc.uam.mx; 6 Centro Universitario Amecameca, Universidad Autónoma del Estado de México, Amecameca CP 56900, Mexico

**Keywords:** feed additive, meta-regression, growth promoter, carcass traits

## Abstract

This study aimed to evaluate the effects of dietary supplementation with guanidinoacetic acid (GAA) on growth performance, antioxidant status, nutrient digestibility, ruminal fermentation, and meat quality in lambs through a meta-analysis. The electronic databases Scopus, Web of Science, ScienceDirect, and Google Scholar were used to identify the articles required for the meta-analysis using the Preferred Reporting Elements for Systematic Reviews and Meta-Analyses (PRISMA) methodology. The data used in this meta-analysis were obtained from 12 peer-reviewed English-language articles published in the last decade (January 2016 to April 2026). All data were analyzed using random-effects models. Dietary supplementation with GAA decreased the feed conversion ratio (*p* < 0.05) and increased (*p* < 0.05) dry matter intake, average daily gain, carcass weight and yield, and *Longissimus dorsi* muscle area. Dietary supplementation with GAA decreased (*p* < 0.001) serum malondialdehyde concentration and increased (*p* ≤ 0.05) serum concentrations of superoxide dismutase, catalase, glutathione peroxidase, and total antioxidant capacity. Dietary supplementation with GAA decreased (*p* < 0.001) ruminal pH and increased (*p* < 0.05) the digestibility of dry matter, organic matter, neutral detergent fiber, and acid detergent fiber, as well as the ruminal concentrations of total volatile fatty acids, acetate, and butyrate. Dietary supplementation with GAA decreased (*p* < 0.001) drip loss in meat and increased (*p* < 0.001) meat pH and meat protein content. In conclusion, guanidinoacetic acid can be used as a dietary additive to improve growth performance, antioxidant status, nutrient digestibility, ruminal fermentation, and meat quality in lambs.

## Simple Summary

Guanidinoacetic acid is the immediate precursor of creatine and plays an important role in regulating cellular energy metabolism. This study analyzed the results of 12 research studies published between 2016 and 2026 to evaluate the effects of dietary supplementation with guanidinoacetic acid in lambs. The results showed that guanidinoacetic acid improved feed intake, weight gain, and feed utilization efficiency in lambs. Furthermore, guanidinoacetic acid enhances antioxidant capacity in lambs by increasing the concentration of endogenous antioxidant defense enzymes and reducing levels of compounds associated with cellular damage. Guanidinoacetic acid promotes feed digestibility and improves the production of volatile fatty acids in the rumen. In meat, guanidinoacetic acid reduces fluid loss and increases protein content. Taken together, these findings indicate that guanidinoacetic acid is a promising additive for improving productivity, health, and meat quality in lambs.

## 1. Introduction

According to Jiang and Wang [1], consumer interest in lamb and goat meat has increased in recent years due to their rich nutritional value and distinctive flavors. To increase the availability of lamb meat in the market, improvements in lamb production parameters are needed [2]. Some beta-adrenergic agonists (e.g., zilpaterol) and dietary antibiotics were used for several years to improve productive performance in small ruminants, particularly lambs [3]. However, the use of these products in animal feed has been restricted in recent years due to the risk of residues in meat, which can negatively affect the health of human consumers [4]. Tannins [5], probiotics and prebiotics [3], curcumin [2], essential oils [6], and guanidinoacetic acid [7] are some feed additives that in recent years have shown high potential as growth promoters of lambs without apparent risk of toxic residues in the meat.

Guanidinoacetic acid (GAA; also known as guanidinoacetate or glycocyamine) is an intermediate metabolite that can be synthesized in the kidneys of lambs and other farm animals from glycine and L-arginine [8]. According to Soares et al. [9], GAA serves as a substrate for creatine biosynthesis in ruminants. Creatine is essential for optimal ruminant growth because it serves as an energy substrate for skeletal muscle development [10]. In beef cattle fed high-forage diets, dietary supplementation with GAA improves nitrogen metabolism and volatile fatty acid production in the rumen [10]. In beef cattle fed high-concentrate diets, dietary supplementation with GAA improves the nutritional quality of the meat [11], as well as nutrient digestibility and antioxidant status in blood serum [10].

Recently, several studies have evaluated the effects of dietary supplementation with GAA on growth performance [12,13], serum antioxidant status [7,14], nutrient digestibility [15,16], ruminal fermentation [11,17], and meat quality [18,19] in lambs. However, the variability in results across studies makes it difficult to draw meaningful conclusions about the effectiveness of GAA as a dietary additive for lambs. For example, López-Aguirre et al. [12] observed that dietary supplementation with high doses (2000 mg/kg DM) of GAA has no significant effects on growth performance and meat quality in lambs. On the other hand, Zhang et al. [11] observed that dietary supplementation with GAA (1500 mg/kg DM) decreases ruminal propionate concentration without negatively affecting growth performance. In contrast, other studies [7,14] reported that dietary supplementation with low doses (≤1000 mg/kg DM) of GAA improves growth performance, serum antioxidant status, and ruminal propionate and total volatile fatty acid production in lambs. According to some authors [8,20,21], the type of GAA (standard or coated), the GAA dose (mg/kg DM), the days of GAA supplementation, and the amount (g/kg DM) of concentrate used in the experimental diets are factors related to the variability of the effects of GAA as a dietary additive for cattle.

Some recent narrative reviews [8,22] provide scientific evidence on the effects of GAA on the metabolism and productivity of some farm animals. However, none of these narrative reviews examined the effects of GAA in detail in lambs or other ruminants. According to some authors [20,23], narrative reviews are considered to have low scientific rigor because their results are often biased by the reviewers’ subjectivity. In contrast, a meta-analysis increases the reliability of results by increasing the number of observations of a treatment’s effect and statistically combining the results reported in several comparable studies [24]. Recently, Gao et al. [20] used meta-analytic statistical procedures to evaluate the effects of dietary GAA supplementation in broiler chickens. However, to date, no scientific manuscripts have evaluated the effects of dietary GAA supplementation in lambs using meta-analysis. Therefore, this study aimed to evaluate the effects of dietary supplementation with guanidinoacetic acid on growth performance, antioxidant status, nutrient digestibility, ruminal fermentation, and meat quality in lambs through meta-analysis.

## 2. Materials and Methods

### 2.1. Literature Search

**Figure 1:**
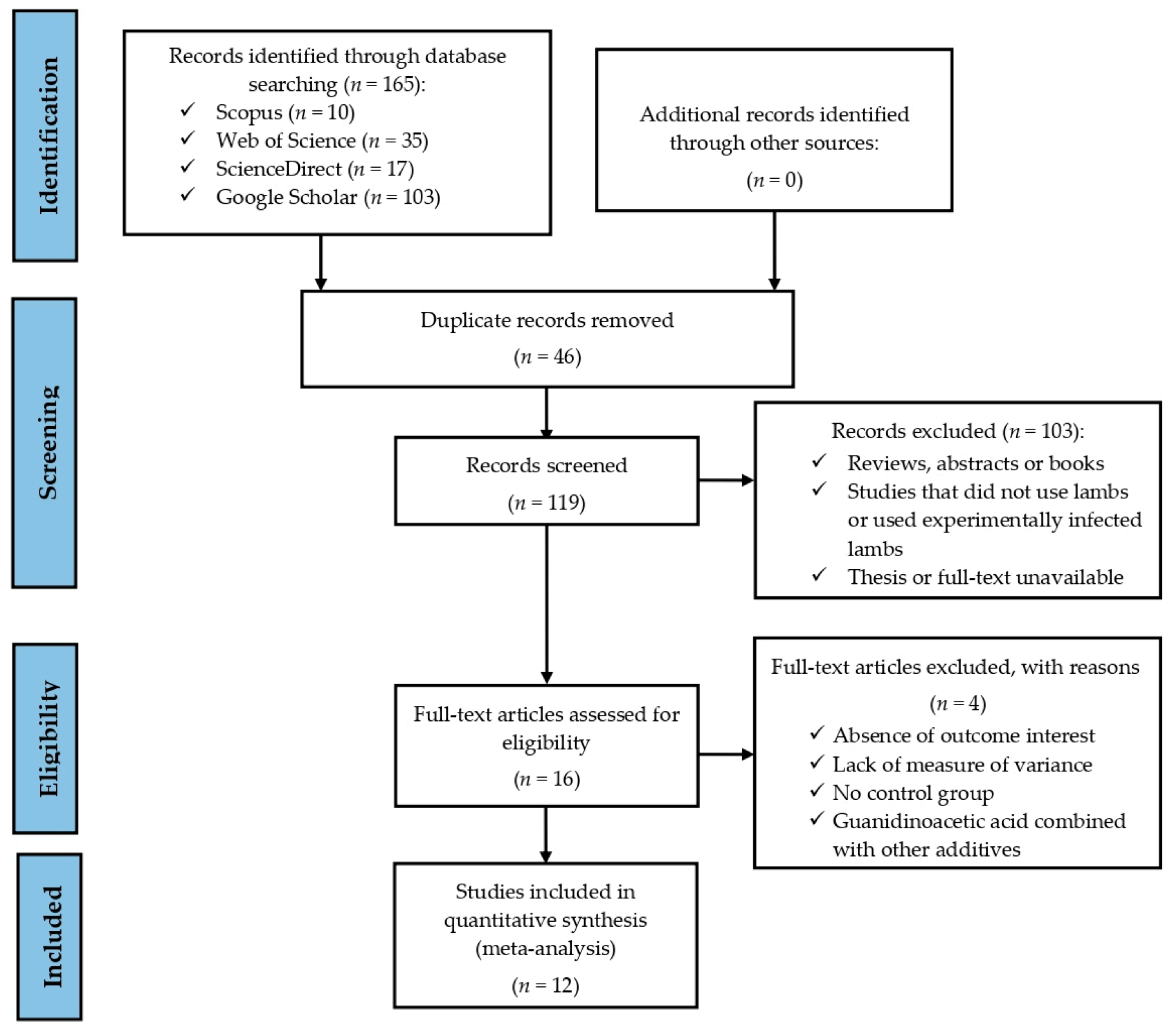
A PRISMA flow diagram detailing the literature search strategy and study selection for the meta-analysis.

The PICO (Population, Intervention, Comparison, and Outcome) format proposed by Hooijmans et al. [25] was used to formulate the research question, which included the following components: lambs as the population (P), dietary supplementation with guanidinoacetic acid as the intervention (I), diets with and without guanidinoacetic acid as the comparison (C), and the outcomes (O) as treatment means related to growth performance, serum antioxidant status, ruminal fermentation, nutrient digestibility, or meat quality. Information was obtained through systematic searches based on the PRISMA guidelines (Preferred Reporting Elements for Systematic Reviews and Meta-Analyses) [26]. The search encompassed scientific documents published in English between January 2016 and april 2026 in Scopus, Google Scholar, Web of Science, and ScienceDirect (Figure 1). The words used in the searches were “lambs”, “guanidinoacetic acid”, “growth performance”, “antioxidant status”, “oxidative state”, “ruminal parameters”, “ruminal fermentation”, “nutrient digestibility”, and “meat quality”.

### 2.2. Eligibility Criteria

The database used in the statistical analyses of this study included 12 scientific articles (Table A1), which met the following criteria: (1) scientific articles that used healthy lambs (not experimentally infected with parasites, bacteria, or viruses) as experimental animals; (2) scientific articles published between January 2016 and April 2026 in English; (3) scientific articles that tested a control diet (without guanidinoacetic acid) against at least one diet supplemented with guanidinoacetic acid; and (4) scientific articles that reported the number of replicates (n), treatment means, and standard error of the mean (SEM) for the response variables of interest (growth performance, serum antioxidant status, ruminal fermentation, nutrient digestibility, and meat quality). Review articles, conference proceedings, theses, and books were not included in the database, as suggested by Borenstein et al. [27].

### 2.3. Data Extraction

From the 12 articles listed in Table A1, n, SEM, and treatment means were extracted for the following groups of response variables: (1) average daily gain (ADG), dry matter intake (DMI), feed conversion ratio (FCR), hot carcass weight (HCW), hot carcass yield (HCY), and *Longissimus dorsi* muscle area (LDMA); (2) serum concentration of superoxide dismutase (SOD), catalase (CAT), glutathione peroxidase (GSH-Px), total antioxidant capacity (T-AOC), and malondialdehyde (MDA); (3) dry matter digestibility (DMD), organic matter digestibility (OMD), crude protein digestibility (CPD), ether extract digestibility (EED), neutral detergent fiber digestibility (NDFD), and acid detergent fiber digestibility (ADFD). (4) Ruminal pH, ruminal ammonia nitrogen (NH_3_-N) concentration, ruminal concentration of total volatile fatty acids (TVFA), and ruminal concentration of acetate, propionate, and butyrate; (5) meat pH at 24 h, lightness (L*), redness (a*), yellowness (b*), drip loss (DL), and moisture, protein, fat, and ash content. Additionally, the following information was extracted from each article: reference (author and year of publication), type of guanidinoacetic acid (standard or coated), guanidinoacetic acid dose (mg/kg DM), guanidinoacetic acid supplementation period (days), and concentrate level (g/kg DM) included in the experimental diets used. During the creation of the meta-analysis database, it was verified that all included datasets from different articles were independent and that there was no overlap in any response variable.

### 2.4. Statistical Analysis, Heterogeneity and Publication Bias

The random-effects model of Der-Simonian and Laird [28] was used to estimate the effect size of dietary supplementation with GAA, using the specialized package “metafor” in R version 4.4.2, as described in detail by Viechtbauer [29]. Weighted mean differences (WMDs) were used to report the results. Comparisons of the GAA-added diet treatments were performed against control diets that had exactly the same characteristics, except for the presence of GAA. Furthermore, potential heterogeneity among the results of the different studies was assessed using meta-regression and subsequently with subgroup analysis, as suggested by Borenstein et al. [27]. Cochran’s Q test and the I^2^ statistic were used to test for and quantify heterogeneity between studies [5,30], where *p* values ≤ 0.05 in the Q test together with I^2^ values > 50% indicated significant heterogeneity [2,31]. Begg’s adjusted rank correlation test [32] and Egger’s regression test [33] were used to assess the possibility of publication bias in the current meta-analysis. These two tests were considered statistically significant when *p* ≤ 0.05.

### 2.5. Meta-Regression and Subgroup Analysis

The effect of the level of concentrate added to the experimental diets (g/kg DM), the period of GAA supplementation (≤60 and >60 days), the type of GAA used (standard or coated), and the GAA dose (<1000 mg/kg of DM and ≥1000 mg/kg of DM) were tested as covariates to assess their contribution to the heterogeneity observed in DMI, ADG, and FCR using meta-regression analyses, as described by Der-Simonian and Laird [28]. The breed of lambs and the geographical origin (country) of the studies included in the database were also tested as covariates in the meta-regression analyses. Subgroup analyses were conducted to evaluate the effects of significant covariates on DMI, ADG, and FCR, as recommended by Koricheva et al. [31] and Hernández-García et al. [23].

## 3. Results

### 3.1. Growth Performance

Table 1 shows that dietary supplementation with GAA increased (*p* < 0.05) DMI, ADG, HCW, HCY, and LDMA. In contrast, a lower (*p* < 0.05) FCR was observed in response to dietary supplementation with GAA.

### 3.2. Antioxidant Status

Dietary supplementation with GAA increased (*p* ≤ 0.05) serum concentrations of SOD, CAT, GSH-Px, and T-AOC (Table 2). In contrast, a lower (*p* < 0.001) serum concentration of MDA was observed in response to dietary supplementation with GAA.

### 3.3. Nutrient Digestibility and Ruminal Fermentation

Table 3 shows that dietary supplementation with GAA increased (*p* < 0.05) DMD, OMD, NDFD, ADFD, NH_3_-N, TVFA, acetate, and butyrate. However, CPD, EED, and ruminal propionate concentration were not affected (*p* > 0.05) by dietary GAA supplementation. Furthermore, dietary GAA supplementation decreased ruminal pH (*p* < 0.001).

### 3.4. Meat Quality

Dietary GAA supplementation increased (*p* < 0.001) meat pH at 24 h and meat protein content (Table 4). However, meat color (L*, a*, and b*) and moisture, fat, and ash content were not affected (*p* > 0.05) by dietary GAA supplementation. Additionally, dietary GAA supplementation decreased DL (*p* < 0.001).

### 3.5. Publication Bias and Meta-Regression

There were no significant effects (*p* > 0.05) in the Egger and Begg tests (Table 1, Table 2, Table 3 and Table 4), indicating that there was no publication bias. However, these results should be interpreted with caution since Begg and Egger’s tests have low power in meta-analyses with a small number of comparisons [27].

Table 1 shows heterogeneity (*p* < 0.001) in DMI, ADG, and FCR. However, there was no heterogeneity (*p* > 0.05) in other response variables related to antioxidant status (Table 2), nutrient digestibility and ruminal fermentation (Table 3), or meat quality (Table 4).

The period of GAA supplementation and the level of concentrate included in the diets did not explain (*p* > 0.05) the heterogeneity observed in DMI, ADG, or FCR (Table 5). However, the doses of GAA included in the diets explained (*p* < 0.05) 17.07%, 50.04%, and 32.59% of the heterogeneity observed in DMI, ADG, and FCR, respectively. Likewise, the type of GAA added to the diets explained (*p* < 0.05) 25.17%, 45.07%, and 29.00% of the heterogeneity observed in DMI, ADG, and FCR, respectively.

### 3.6. Subgroup Analysis

**Figure 2:**
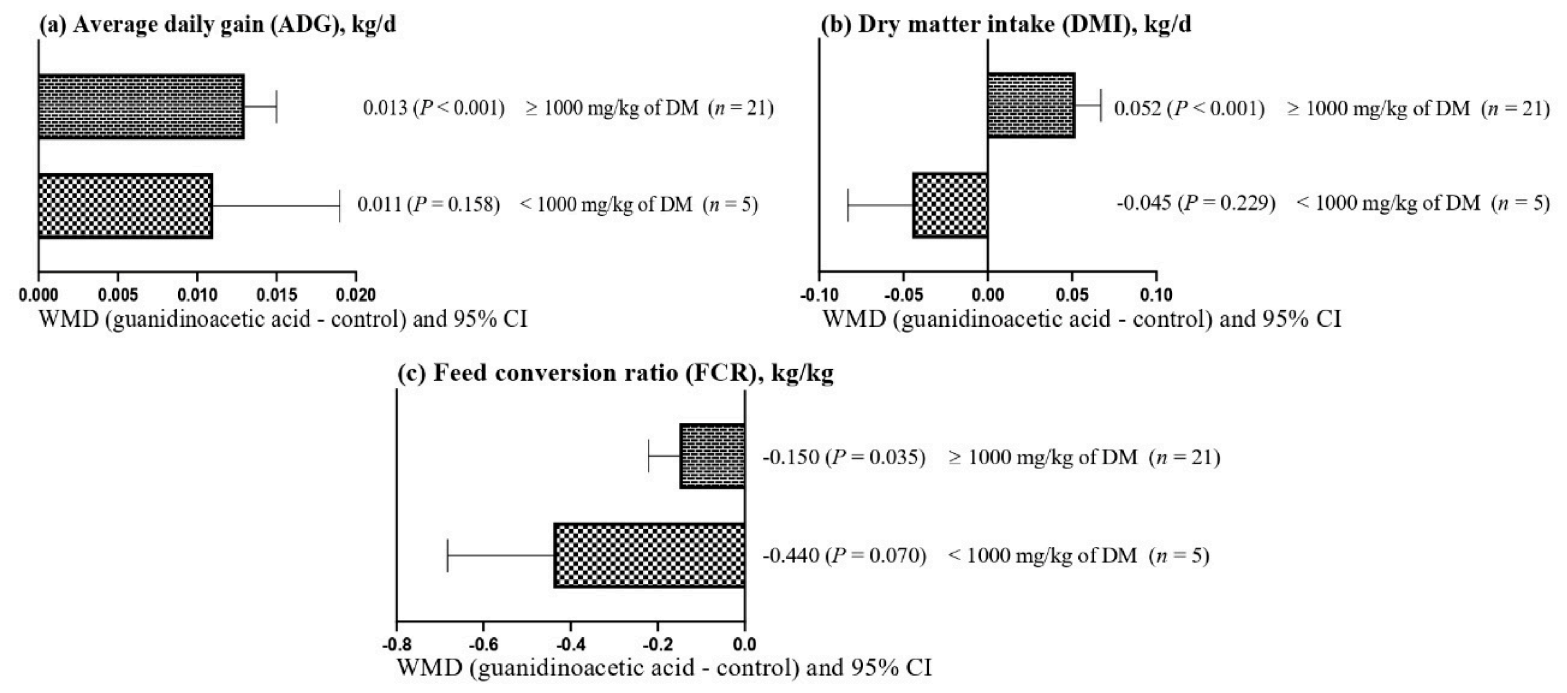
Subgroup analysis (subgroup = guanidinoacetic acid dose (mg/kg of DM) of the effect of including guanidinoacetic acid in the diets of lambs, WMD = weighted mean differences between guanidinoacetic acid treatments and control.

Figure 2a shows that ADG increased (*p* < 0.001) with high doses (≥1000 mg/kg of DM) of GAA; however, low doses (<1000 mg/kg of DM) of GAA did not affect ADG (*p* > 0.05). Similarly, DMI increased (*p* < 0.001) when high doses (≥1000 mg/kg of DM) of GAA were used; however, low doses (<1000 mg/kg of DM) of GAA did not affect DMI (*p* > 0.05; Figure 2b). Figure 2c shows that FCR decreased (*p* < 0.001) with high doses (≥1000 mg/kg of DM) of GAA; however, low doses (<1000 mg/kg of DM) of GAA did not affect (*p* > 0.05) FCR.

**Figure 3:**
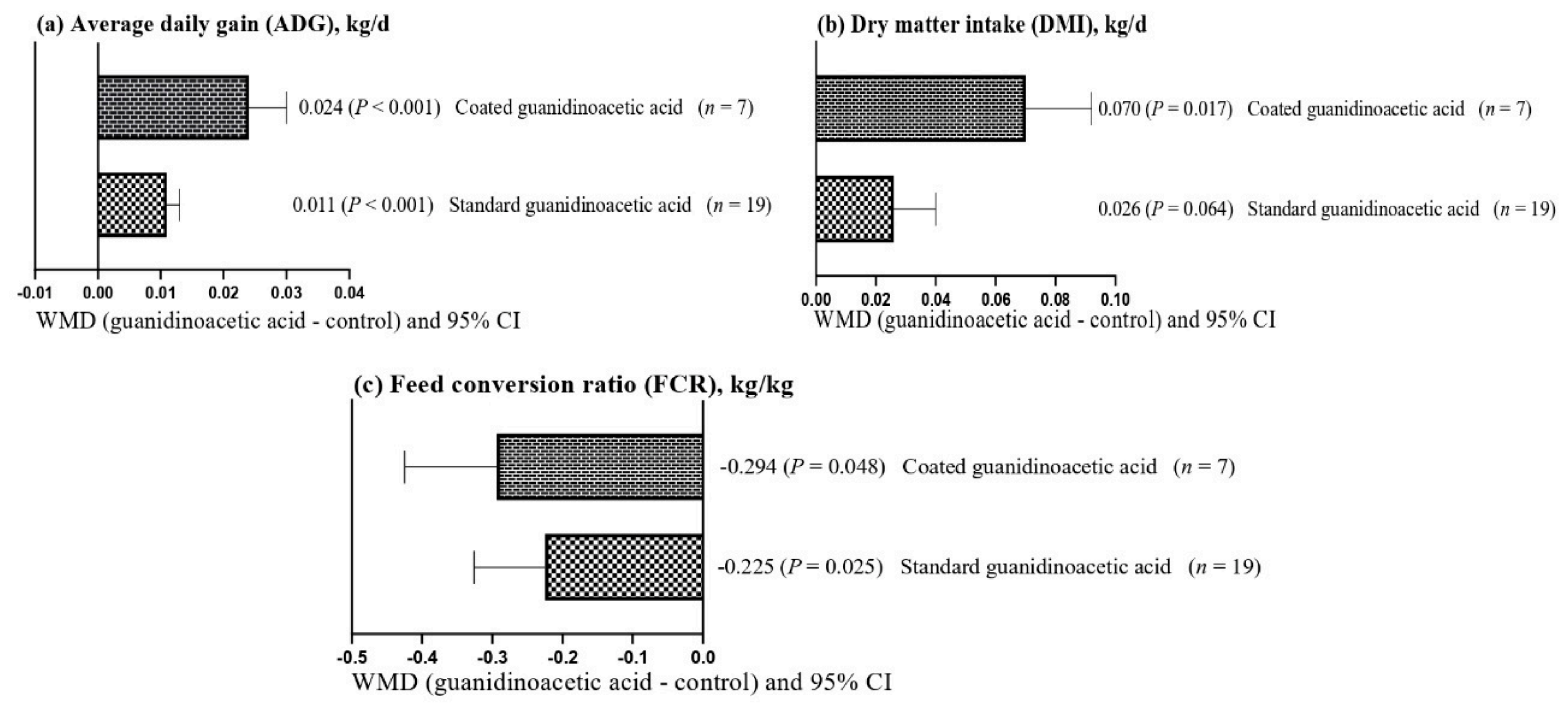
Subgroup analysis (subgroup = type of guanidinoacetic acid) of the effect of including guanidinoacetic acid in the diets of lambs, WMD = weighted mean differences between guanidinoacetic acid treatments and control.

Figure 3a shows that ADG increased (*p* < 0.001) regardless of the type of GAA included in the diets. However, the effect size was larger with coated GAA (WMD = 0.024 kg/d) than with standard GAA (WMD = 0.011 kg/d). DMI increased (*p* < 0.05) when coated GAA was used; however, DMI was not affected (*p* > 0.05) when standard GAA was included in the diets (Figure 3b). Figure 3c shows that FCR decreased (*p* < 0.05) regardless of the type of GAA included in the diets. However, the effect size was larger (WMD = −0.294 kg/kg) with coated GAA than with standard GAA (WMD = −0.225 kg/kg).

## 4. Discussion

### 4.1. Growth Performance

The higher DMI and ADG values observed in the current meta-analysis indicate that dietary supplementation with GAA stimulates voluntary feed intake and growth rate in lambs. These findings are consistent with those reported by Liu et al. [34], who observed higher DMI (+6.9%) and ADG (+26.7%) in finishing beef cattle supplemented with GAA. In the present study, GAA increased OMD, which could explain the observed increase in DMI, as there is a strong positive correlation (r = 0.73 to 0.87) between OMD and DMI in ruminants [35]. Furthermore, the higher DMI could be related to the higher NDFD obtained with GAA in the current meta-analysis, since, according to Zapata et al. [3], when the NDFD is high, the ruminal retention time of the feed and the effect of physical satiety decrease, which stimulates voluntary feed intake and results in higher DMI. The increase in NDFD promotes greater production of volatile fatty acids, which explains the slight decrease in ruminal pH observed in the current meta-analysis. Since this decrease in ruminal pH remained within the physiological range (6.4–6.8) compatible with the activity of cellulolytic ruminal bacteria, it does not compromise fiber digestion and reflects more efficient ruminal fermentation [35].

The higher ADG observed in lambs supplemented with GAA in the current meta-analysis is consistent with the results reported by Yi et al. [10], who observed higher ADG (+16.4% to +24.8%) in beef cattle supplemented with GAA. Furthermore, the lower FCR observed in this meta-analysis with GAA use suggests that lambs supplemented with this additive had greater feed efficiency. Similarly, other authors [36] observed lower FCR (−24.5% to −34.9%) in beef cattle supplemented with GAA. The higher ADG and lower FCR values obtained in the current meta-analysis using GAA as a dietary additive could be explained by higher DMD, OMD, and NDFD, as well as by the higher ruminal TVFA concentration observed in response to GAA supplementation. In addition, dietary supplementation with GAA increases (+37.7%) ruminal microbial protein synthesis [17], which leads to a greater supply of amino acids to the duodenum that can be used to form new body tissues and improve ADG and FCR. On the other hand, dietary supplementation with various levels (500 to 1500 mg/kg DM) of GAA increases (+14.2% to +35.4%) the serum concentration of insulin-like growth factor 1 [11,17,37], which, according to Deori et al. [38], is positively correlated (r = 0.541) with ADG in small ruminants. In addition, Li et al. [39] observed that GAA decreases (−32.0% to −75.5%) the presence of Rikenellaceae rumen bacteria, which have a negative correlation (r = −0.76) with ADG and feed efficiency in lambs [35].

In the current meta-analysis, the higher HCW, HCY, and LDMA values obtained in lambs supplemented with GAA may have a positive impact since, according to Kwon et al. [40], these three parameters (HCW, HCY, and LDMA) have a strong positive correlation (r = 0.50 to 0.91) with higher yields of meat cuts preferred by consumers, such as loin, ribs, and tenderloin. The higher HCW could be directly related to the higher ADG observed in lambs supplemented with GAA, since, according to Fernandes et al. [41], HCW is positively correlated (r = 0.62) with ADG in ruminants. Likewise, the higher HCY could be explained by the higher LDMA observed in lambs supplemented with GAA, due to the strong positive correlation (r = 0.74) between LDMA and HCY in ruminants [42]. On the other hand, Li et al. [39] reported that GAA intake increases (+53.7% to +58.5%) the expression of the mTOR gene (mammalian target of rapamycin) in the *Longissimus dorsi* muscle of lambs. In lambs, mTOR stimulates anabolic processes and inhibits catabolic ones [2], which could directly contribute to the higher LDMA value in the GAA-supplemented lambs in the present meta-analysis. Furthermore, Li et al. [18] reported that GAA intake increases the diameter of muscle fibers in the *Longissimus dorsi* muscle of lambs, which would explain the higher LDMA observed in the current meta-analysis.

### 4.2. Antioxidant Status

In sheep production systems, several environmental (heat stress), dietary (oxidized fat), and physiological factors, such as high growth rates, stimulate the overproduction of reactive oxygen species (ROS) in animal cells [43]. The accumulation of ROS in lamb cells leads to oxidative stress (OS) [23], and, according to Macías-Cruz et al. [43], OS negatively impacts the health, welfare, growth performance, and meat quality of lambs. According to some authors [10,44], dietary supplementation with GAA could improve antioxidant status in ruminant blood serum, as GAA is a precursor of creatine synthesis and can neutralize ROS. In the present meta-analysis, dietary supplementation of GAA decreased MDA and increased SOD, CAT, GSH-Px, and T-AOC. Similar to the results of the present meta-analysis, Yi et al. [10] also observed lower serum MDA concentrations and higher serum levels of SOD, CAT, GSH-Px, and T-AOC in beef cattle supplemented with GAA.

MDA is a byproduct of lipid peroxidation, and low serum levels indicate low ROS-induced damage to cell membrane phospholipids [2,23]. Furthermore, the observed reduction in serum MDA concentration in lambs supplemented with GAA could have a positive impact on productivity, since, according to Macías-Cruz et al. [43], serum MDA concentration has a negative correlation (r = −0.55) with ADG in lambs. On the other hand, elevated levels of SOD, CAT, GSH-Px, and T-AOC simultaneously indicate robust ROS scavenging activity at the cellular level [7]. In lambs, SOD converts superoxide ($O_{2}^{-}$) to hydrogen peroxide (H_2_O_2_), while CAT and GSH-Px neutralize H_2_O_2_ molecules into H_2_O and O_2_ [2,18].

Based on the reviewed scientific literature, the exact mechanism by which GAA increases serum levels of SOD, CAT, and GSH-Px in lambs remains unclear. However, it has been suggested that GAA could increase the endogenous production of SOD, CAT, and GSH-Px by activating the erythroid nuclear factor 2-related factor 2 (Nrf2) signaling pathway [7], since increased Nrf2 activity leads to greater production of SOD, CAT, and GSH-Px in lambs [2,10]. Furthermore, the increase in serum T-AOC levels observed in the present study could be mainly explained by the increases in SOD, CAT, and GSH-Px in lambs supplemented with GAA.

### 4.3. Nutrient Digestibility and Rumen Fermentation

Dietary supplementation with GAA improved DMD, OMD, NDFD, and ADFD. Similarly, other authors [10,34] reported significantly higher values (+10.7% to +15.8%) for DMD, OMD, NDFD, and ADFD in beef cattle supplemented with GAA. A recent study [34] reported that dietary supplementation with GAA improves the relative abundance of fungi (+11.5%) and bacteria, including *Fibrobacter succinogenes* (+14.2%), *Ruminococcus albus* (+45.3%), and *Ruminococcus flavefaciens* (+56.2%). These mechanisms of action of GAA in ruminants could explain the positive effects observed in DMD, OMD, NDFD, and ADFD of the present meta-analysis, since, according to Weimer et al. [45], when the ruminal concentration of fungi, *F. succinogenes*, *R. albus*, and *R. flavefaciens* is high, the values of DMD, OMD, NDFD, and ADFD increase.

In the current study, dietary supplementation with GAA decreased ruminal pH and increased ruminal NH_3_-N concentration. However, the mean ruminal pH values observed in the current meta-analysis in lambs supplemented with GAA are within the range of ruminal pH considered physiologically normal (6.4 to 6.8) in healthy small ruminants [46]. This suggests that GAA could be used as a dietary additive for lambs without negatively affecting ruminal homeostasis. On the other hand, dietary supplementation with GAA increases (+10.1%) the concentration of protozoa in the ruminal fluid of lambs [11]. Rumen protozoa can ingest significant amounts of protein present in the rumen contents, hydrolyze them, and release NH_3_-N, which would explain the higher rumen concentration of NH_3_-N observed in lambs supplemented with GAA in the current meta-analysis. Furthermore, in lambs, GAA increases (+27.9% to +78.7%) the relative abundance of rumen bacteria of the genus *Prevotella* [39], which, according to Yi et al. [47], are positively correlated (r = 0.63) with rumen NH_3_-N production and concentration.

The higher TVFA concentration observed in the current study could be explained by the higher dry matter intake (DMI) of the lambs that received GAA, since, according to Sun et al. [48], when the DMI of small ruminants is high, their ruminal TVFA production increases because there is more organic matter available for fermentation in the rumen. Furthermore, the higher TVFA concentration could be directly related to the increase observed in the ruminal concentration of acetate and butyrate in the GAA-supplemented lambs. On the other hand, dietary supplementation with GAA increases (+32.6% to +56.1%) the relative abundance of bacteria of the genera *Ruminococcus* and *Butyrivibrio* in the ruminal fluid of lambs and beef cattle [34,39]. These mechanisms of action of GAA would explain the higher ruminal concentration of acetate and butyrate observed in the current meta-analysis because, according to some authors [49,50], the main bacterial species that produce acetate and butyrate are found within the genera *Ruminococcus* and *Butyrivibrio*, respectively.

### 4.4. Meat Quality

Dietary supplementation with GAA increased the pH in lamb meat. Despite the observed increase, the pH of lamb meat in both groups (control and GAA-supplemented) had values lower than 5.8, which is considered within the normal range for sheep meat [51]. On the other hand, Ke et al. [52] indicate that, in lamb meat, color is a parameter that directly influences consumers’ perception of its quality. In the current study, dietary supplementation with GAA did not affect the color (L*, a*, and b*) of the meat, suggesting that GAA can be used as a dietary additive for lambs without compromising the meat’s appearance and quality. Furthermore, in this meta-analysis, dietary supplementation with GAA reduced DL, indicating that GAA improves juiciness in lamb meat. GAA intake increases (+19.8% to +55.4%) the activity of SOD, CAT, and GSH-Px enzymes in lamb muscle tissue [18]. Increased simultaneous activity of these three antioxidant enzymes (SOD, CAT, and GSH-Px) decreases protein carbonylation [23]. This indirect effect of GAA would explain the lower DL observed in the current meta-analysis, as when protein carbonylation is low, DL in lamb meat decreases [53]. Furthermore, GAA intake can increase osmotic pressure in muscle cells by increasing their creatine and phosphocreatine content [19]. This effect could improve water-holding capacity in lamb meat and lead to a lower DL, as suggested by other authors [52].

In the present meta-analysis, dietary supplementation with GAA increased meat protein content without affecting moisture, fat, or ash content, which could be beneficial for lamb consumers. Similarly, Zhang et al. [21] observed that dietary supplementation with GAA increased protein content in beef cattle without affecting moisture, ether extract, or ash. In lambs, dietary supplementation with GAA increases the expression levels of the PI3K-Akt signaling pathway and the phosphorylation of the FoxO1 gene in the *Longissimus dorsi* muscle [18]. These effects could be related to the higher protein content observed in the meat of GAA-supplemented lambs in the current meta-analysis, since, according to Yi et al. [10], increased PI3K-Akt activity decreases proteolytic degradation by phosphorylating FoxO1 and promotes the synthesis of new proteins.

## 5. Conclusions

Dietary supplementation with guanidinoacetic acid can serve as a nutritional strategy to replace beta-adrenergic agonists or dietary antibiotics as growth promoters, as it improves growth rate, feed efficiency, carcass yield, and *Longissimus dorsi* muscle area without adversely affecting lamb meat quality. Furthermore, dietary supplementation with guanidinoacetic acid enhances the activity of antioxidant enzymes such as catalase, superoxide dismutase, and glutathione peroxidase, while simultaneously improving the digestibility of dry matter, neutral detergent fiber, and acid detergent fiber, as well as ruminal acetate and butyrate production. However, these results should be interpreted with caution, as the productive variables had high heterogeneity, and in most of the response variables evaluated, the database included a limited number of studies (≤12) and comparisons (≤26).

## Figures and Tables

**Table 1:** Growth performance and carcass traits of growing lambs supplemented with guanidinoacetic acid.

Item	NC				Heterogeneity		Begg Test ^2^	Egger Test ^3^
		Control Means (SD)	WMD (95% CI)	*p*-Value	I^2^ (%)	*p*-Value ^1^	*p*-Value	*p*-Value
DMI, kg/d	26	1.180 (0.227)	0.039 (0.013; 0.066)	0.004	87.43	<0.001	0.841	0.532
ADG, kg/d	26	0.215 (0.063)	0.014 (0.009; 0.019)	<0.001	67.29	<0.001	0.247	0.086
FCR, kg/kg	26	6.26 (2.11)	−0.224 (−0.378; −0.069)	0.005	64.08	<0.001	0.134	0.060
HCW, kg	9	18.386 (5.034)	1.010 (0.366; 1.653)	0.002	42.54	0.071	0.433	0.245
HCY, %	9	45.962 (2.769)	1.864 (1.342; 2.387)	<0.001	39.68	0.103	0.487	0.173
LDMA, cm^2^	9	18.744 (5.191)	2.750 (1.970; 3.530)	<0.001	49.15	0.065	0.216	0.152

NC: number of comparisons between treatments supplemented with guanidinoacetic acid and control; SD: standard deviation; WMD: weighted mean differences between treatments supplemented with guanidinoacetic acid and control; CI: confidence interval of WMD; I^2^: proportion of total variation in size effect estimates that is due to heterogeneity; ^1^ *p*-value to Cochran’s Q statistic; ^2^: Begg’s adjusted rank correlation; ^3^: Egger’s regression asymmetry test; DMI: dry matter intake; ADG: average daily gain; FCR: feed conversion ratio; HCW: hot carcass weight; HCY: hot carcass yield; LDMA: *Longissimus dorsi* muscle area.

**Table 2:** Antioxidant status in the blood serum of growing lambs supplemented with guanidinoacetic acid.

Item	NC				Heterogeneity		Begg Test ^2^	Egger Test ^3^
		Control Means (SD)	WMD (95% CI)	*p*-Value	I^2^ (%)	*p*-Value ^1^	*p*-Value	*p*-Value
SOD, U/mL	13	72.37 (18.72)	37.804 (23.230; 52.378)	<0.001	36.14	0.110	0.063	0.060
CAT, U/mL	9	7.95 (0.62)	1.456 (−0.064; 2.976)	0.050	0.00	0.989	0.165	0.115
GSH-Px, U/mL	13	608.03 (236.13)	56.486 (33.630; 79.342)	<0.001	33.26	0.52	0.182	0.392
T-AOC, U/mL	10	9.93 (2.81)	1.489 (0.869; 2.109)	<0.001	48.78	0.061	0.772	0.942
MDA, nmol/mL	13	4.74 (0.87)	−1.036 (−1.413; −0.658)	<0.001	41.35	0.085	0.567	0.298

NC: number of comparisons between treatments supplemented with guanidinoacetic acid and control; SD: standard deviation; WMD: weighted mean differences between treatments supplemented with guanidinoacetic acid and control; CI: confidence interval of WMD; I^2^: proportion of total variation in size effect estimates that is due to heterogeneity; ^1^ *p*-value to Cochran’s Q statistic; ^2^: Begg’s adjusted rank correlation; ^3^: Egger’s regression asymmetry test; SOD: superoxide dismutase; CAT: catalase; GSH-Px: glutathione peroxidase; T-AOC: total antioxidant capacity; MDA: malondialdehyde.

**Table 3:** Nutrient digestibility and ruminal fermentation of growing lambs supplemented with guanidinoacetic acid.

Item	NC				Heterogeneity		Begg Test ^2^	Egger Test ^3^
		Control Means (SD)	WMD (95% CI)	*p*-Value	I^2^ (%)	*p*-Value ^1^	*p*-Value	*p*-Value
Nutrient digestibility, g/100 g								
DMD	10	73.77 (5.37)	1.721 (1.186; 2.257)	<0.001	11.39	0.338	0.772	0.467
OMD	10	76.50 (6.59)	1.503 (1.004; 2.003)	<0.001	0.00	0.719	0.289	0.180
CPD	10	75.41 (4.72)	0.593 (−0.042; 1.227)	0.067	0.00	0.654	0.148	0.568
EED	9	83.01 (4.42)	0.372 (−0.517; 1.260)	0.412	0.00	0.997	0.817	0.481
NDFD	10	46.54 (1.72)	2.262 (1.328; 3.196)	<0.001	46.14	0.062	0.629	0.558
ADFD	10	38.96 (3.17)	2.163 (0.809; 3.517)	0.002	41.17	0.084	0.923	0.689
Ruminal fermentation								
Ruminal pH	12	6.62 (0.26)	−0.062 (−0.090; −0.035)	<0.001	0.00	0.916	0.286	0.219
NH_3_-N, mg/dL	12	12.06 (4.80)	0.432 (0.247; 0.616)	<0.001	0.00	9.76	0.092	0.073
TVFA mM	12	86.06 (6.63)	5.634 (3.294; 7.974)	<0.001	39.85	0.068	0.721	0.464
SCFA, mol/100 mol								
Acetate	12	56.72 (3.65)	0.334 (0.091; 0.577)	0.007	20.82	0.239	0.878	0.651
Propionate	12	29.97 (6.28)	0.290 (−0.178; 0.758)	0.224	48.98	0.129	0.139	0.235
Butyrate	12	10.89 (2.91)	0.415 (0.031; 0.800)	0.034	41.32	0.306	0.878	0.596

NC: number of comparisons between treatments supplemented with guanidinoacetic acid and control; SD: standard deviation; WMD: weighted mean differences between treatments supplemented with guanidinoacetic acid and control; CI: confidence interval of WMD; I^2^: proportion of total variation in size effect estimates that is due to heterogeneity; ^1^ *p*-value to Cochran’s Q statistic; ^2^: Begg’s adjusted rank correlation; ^3^: Egger’s regression asymmetry test; DMD: dry matter digestibility; OMD: organic matter digestibility; CPD: crude protein digestibility; EED: ether extract digestibility; NDFD: neutral detergent fiber digestibility; ADFD: acid detergent fiber digestibility; NH_3_-N: ammonia nitrogen; TVFA: total volatile fatty acids.

**Table 4:** Meat quality of growing lambs supplemented with guanidinoacetic acid.

Item	NC				Heterogeneity		Begg Test ^2^	Egger Test ^3^
		Control Means (SD)	WMD (95% CI)	*p*-Value	I^2^ (%)	*p*-Value ^1^	*p*-Value	*p*-Value
Meat pH 24 h	9	5.41 (0.17)	0.120 (0.068; 0.171)	<0.001	0.00	0.898	0.737	0.677
Lightness (L*)	6	33.13 (5.40)	−0.263 (−1.020; 0.493)	0.495	0.00	0.484	0.821	0.638
Redness (a*)	6	16.00 (0.36)	0.246 (−0.187; 0.679)	0.265	32.21	0.194	0.822	0.552
Yellowness (b*)	6	8.89 (1.77)	−0.184 (−0.443; 0.076)	0.166	0.00	0.519	0.816	0.273
Drip loss (DL), %	6	14.80 (3.16)	−1.236 (−1.724; −0.747)	<0.001	32.94	0.189	0.289	0.275
Meat composition, g/100 g								
Moisture	5	75.82 (0.32)	−0.062 (−0.295; 0.170)	0.599	0.00	0.955	0.157	0.265
Protein	5	20.18 (0.24)	0.646 (0.471; 0.821)	<0.001	0.00	0.536	0.999	0.948
Fat	8	3.51 (1.03)	0.077 (−0.058; 0.212)	0.262	0.00	0.756	0.195	0.072
Ash	5	1.24 (0.16)	0.040 (−0.002; 0.081)	0.062	0.00	0.870	0.999	0.450

NC: number of comparisons between treatments supplemented with guanidinoacetic acid and control; SD: standard deviation; WMD: weighted mean differences between treatments supplemented with guanidinoacetic acid and control; CI: confidence interval of WMD; I^2^: proportion of total variation in size effect estimates that is due to heterogeneity; ^1^ *p*-value to Cochran’s Q statistic; ^2^: Begg’s adjusted rank correlation; ^3^: Egger’s regression asymmetry test.

**Table 5:** Meta-regression comparing the associations between covariates and measured outcomes.

Outcomes	Covariates	QM	Df	*p*-Value	R^2^ (%)
Dry matter intake (DMI)	Supplementation period	0.086	1	0.769	0.00
	Guanidinoacetic acid dose	5.734	1	0.017	17.07
	Type of guanidinoacetic acid	3.062	1	0.040	25.17
	Concentrate level	0.065	1	0.798	0.00
	Breed of lambs	0.317	4	0.260	1.68
	Geographic origin (country)	0.004	1	0.948	0.29
Average daily gain (ADG)	Supplementation period	0.230	1	0.631	0.00
	Guanidinoacetic acid dose	6.847	1	<0.001	50.04
	Type of guanidinoacetic acid	4.516	1	0.034	45.07
	Concentrate level	0.394	1	0.530	0.00
	Breed of lambs	0.736	4	0.728	0.00
	Geographic origin (country)	0.147	1	0.701	1.56
Feed conversion ratio (FCR)	Supplementation period	0.182	1	0.669	0.00
	Guanidinoacetic acid dose	2.638	1	0.039	32.59
	Type of guanidinoacetic acid	3.010	1	0.021	29.00
	Concentrate level	0.737	1	0.391	0.00
	Breed of lambs	8.338	4	0.080	25.05
	Geographic origin (country)	0.438	1	0.508	2.92

QM: coefficient of moderators; QM is considered significant at *p ≤* 0.05; Df: degree of freedom; R^2^: the amount of heterogeneity accounted for.

## Data Availability

The datasets used and analyzed during the current study are available from the corresponding author on reasonable request. The data are not publicly available due to restrictions on privacy.

## Abbreviations

The following abbreviations are used in this manuscript:

GAA	Guanidinoacetic acid
DMI	Dry matter intake
ADG	Average daily gain
FCR	Feed conversion ratio
SOD	Superoxide dismutase
CAT	Catalase
GSH-Px	Glutathione peroxidase
MDA	Malondialdehyde
T-AOC	Total antioxidant capacity
DMD	Dry matter digestibility
OMD	Organic matter digestibility
EED	Ether extract digestibility
NDFD	Neutral detergent fiber digestibility
ADFD	Acid detergent fiber digestibility
NH_3_-N	Ammonia nitrogen
TVFA	Total volatile fatty acids

## Appendix A

**Table A1:** Description of the studies included in the meta-analysis database.

Reference	Type of GAA	Dose, mg/kg DM	SP, Days	Concentrate, g/kg DM	Breed	Geographic Origin (Country)
Jin et al. [14]	Standard GAA	500, 750, 1000	90	400	Hu	China
Li et al. [18]	Standard GAA	900	70	700	Dorper x Small tailed Han	China
Li et al. [15]	Standard GAA, Coated GAA	1000	60	750, 800	Small tailed Han	China
Li et al. [39]	Standard GAA, Coated GAA	1000	60, 120	750, 800	Small tailed Han	China
Li et al. [19]	Standard GAA, Coated GAA	1000	62	750	Small tailed Han	China
López-Aguirre et al. [12]	Standard GAA	2000	60	815	Dorper x Pelibuey	Mexico
Ma et al. [16]	Standard GAA	1500	60	600	Kazakh	China
Ren et al. [17]	Standard GAA	900	60	700	Dorper x Small tailed Han	China
Zhang et al. [13]	Standard GAA	500, 1000, 1500	100	600	Kazakh	China
Zhang et al. [11]	Standard GAA	500, 1000, 1500	45	600	Kazakh	China
Zhu et al. [7]	Standard GAA, Coated GAA	1000	45	600	Hu	China
Zhu et al. [37]	Standard GAA, Coated GAA	1000	45	600	Hu	China

SP: supplementation period; GAA: guanidinoacetic acid; DM: dry matter.
